# Measurement of Gas-Oil Two-Phase Flow Patterns by Using CNN Algorithm Based on Dual ECT Sensors with Venturi Tube

**DOI:** 10.3390/s20041200

**Published:** 2020-02-21

**Authors:** Zhuoqun Xu, Fan Wu, Xinmeng Yang, Yi Li

**Affiliations:** 1 Graduate School at Shenzhen, Tsinghua University, Shenzhen 518055, China; xuzq18@mails.tsinghua.edu.cn (Z.X.); yxm17@mails.tsinghua.edu.cn (X.Y.); 2 Graduate School at Suzhou, University of Science and Technology of China, Suzhou 215000, China; wf18@mail.ustc.edu.cn

**Keywords:** convolutional neural network, oil-gas two-phase flow, electrical capacitance tomography

## Abstract

In modern society, the oil industry has become the foundation of the world economy, and how to efficiently extract oil is a pressing problem. Among them, the accurate measurement of oil-gas two-phase parameters is one of the bottlenecks in oil extraction technology. It is found that through the experiment the flow patterns of the oil-gas two-phase flow will change after passing through the venturi tube with the same flow rates. Under the different oil-gas flow rate, the change will be diverse. Being motivated by the above experiments, we use the dual ECT sensors to collect the capacitance values before and after the venturi tube, respectively. Additionally, we use the linear projection algorithm (LBP) algorithm to reconstruct the image of flow patterns. This paper discusses the relationship between the change of flow patterns and the flow rates. Furthermore, a convolutional neural network (CNN) algorithm is proposed to predict the oil flow rate, gas flow rate, and GVF (gas void fraction, especially referring to sectional gas fraction) of the two-phase flow. We use ElasticNet regression as the loss function to effectively avoid possible overfitting problems. In actual experiments, we compare the Typical-ECT-imaging-based-GVF algorithm and SVM (Support Vector Machine) algorithm with CNN algorithm based on three different ECT datasets. Three different sets of ECT data are used to predict the gas flow rate, oil flow rate, and GVF, and they are respectively using the venturi front-based ECT data only, while using the venturi behind-based ECT data and using both these data.

## 1. Introduction 

Multiphase flow measurement technology is important in the exploitation of petroleum, the accurate measurement of gas and oil flow rate in the oil-gas two phase flow has been the current research issue. In the traditional flow measurement, multiple mixtures that were obtained in oil wells need to be separated in the well for single-phase measurements [1]. This method improves the accuracy of single-phase measurement, but separating multiphase flow is too complicated [2], the equipment is expensive, and the efficiency is low. Therefore, it is necessary to find an accurate and efficient online measuring technology for multiphase flow [3].

Concerning the flow measurement of two-phase flow, there are many technologies that have been used. Mohmmed et al. and Abbagoni and Yeung introduced high-speed cameras (with transparent tube segments) to take high-speed shooting records for convection type [4,5], while Dong et al. used ultrasonic Doppler sensors to estimate the total surface speed of the oil-water two phase flow [6]. P. Aarabi JeshvaghanI et al. proposed and implemented a flow rate measurement method that was based on gamma-ray attenuation to identify temperature independent flow [7]. The content of each phase in the pipe can be measured according to the attenuation of the rays by the fluid in the pipe because different fluids in the same pipe absorb gamma rays differently. AGAR corporation in the United States has designed a low-cost new multi-phase flow meter, AGAR MPFM50, which combines Coriolis technology with traditional flow measurement equipment to achieve better flow measurement results [8]. China’s homer corporation has developed a short-section multiphase flowmeter that uses venturi tube to measure the total flow in a pipe, a multivariate sensor for measuring the temperature and pressure of the fluid as it flows through the venturi tube, and a dual-gamma sensor was used to measure the gas flow rate and water flow rate of the fluid [9]. In addition, commonly used methods include capacitance probe, ultrasonic doppler sensor [10], acoustic emission [11], fiber optic probe [12], wire network sensor [13], and so on. These methods are feasible in a laboratory environment. However, when considering the complexity of the oilfield environment, the application of these methods in real-world environments can be hampered [14]. For example, the transparent pipe segments that are required for high-speed cameras cannot be achieved in practical applications, probes, and wire-mesh sensors that are in contact with fluids in the experiment are difficult to repair in the oil field environment in the case of bad weather [15]. In contrast, electrical capacitance tomography (ECT) has the characteristics of fast measurement speed (up to 5000 frames per second), low application cost, and mature product development. The University of Manchester (Manchester, UK) developed the real-time ECT system in collaboration with the University of Leeds (Leeds, UK) and Schlumberger Cambridge research LTD. In addition, the Morgantown Energy Technology Center (METC) of the US Department of Energy has independently developed the ECT sensor system [16,17]. The system has been successfully applied to the flow measurement of oil-gas two-phase flow in oil field pipelines [18]. Ismail, I. et al. used ECT sensor device to complete the flow measurement of the oil-gas two-phase flow, and completed the cycle test under different flow patterns [18]. Zhang and Wang used ECT sensor technology combined with the artificial neural network to complete the identification of gas-liquid two-phase flow pattern, and completed the measurement of oil flow rate [19]. In brief, the ECT sensor has lower requirement on working environment, and it can achieve accurate measurement and good real-time performance [19]. Hence, ECT is a commonly used detection method in the field of oil fields [20].

The venturi tube is a throttling differential pressure gauge that has been widely used in the flow measurement of single-phase or two-phase flow [21,22]. The submarine multiphase flowmeter, which was developed by Norway’s ROXAR, uses a venturi-tube flowmeter combined with a gamma-ray densitometer to improve the accuracy of flow rates measurements [23]. It has the characteristics of accurate measurement, low energy consumption, stable performance, and convenient maintenance, and it has wide applications in the petrochemical industry [24,25]. Venturi tubes have been widely used in single-stream measurement and multiphase flow measurement. Therefore, the paper uses a combination of venturi tube and dual ECT sensors to observe the flow patterns of the oil-gas two-phase flow before and after the venturi tube, and imaged by LBP (linear projection algorithm) image reconstruction algorithm. 

In the flow of two phase, the interface of two phase is distributed into different geometric shapes or structural forms, which are so-called two-phase flow patterns [26]. It is very complicated to define and classify the flow patterns of oil and gas two-phase flow. Kosterin obtained the classification of different flow patterns according to the form of interfacial phase distribution, and obtained the flow pattern diagram of horizontal pipeline, which was used to describe the flow pattern distribution [27]. In addition, Barnea et al. proposed the flow pattern diagram of the horizontal tube [28], while Caetano et al. proposed the flow pattern diagram of the vertical ascending tube [29]. The current common flow patterns include bubble flow, stratified flow, wavy flow, slug flow, and annular flow [30].

The observation method is the method often used to identify the flow patterns, but this usually leads to the subjectivity of the recognition. Some intelligent algorithms have been proposed to objectively recognize the flow patterns. For instance, wavelet analysis methods [31], support vector machines, genetic algorithms, etc. Marashdeh et al. used the feed-forward neural network and analogue Hopfield network technology for nonlinear image reconstruction of electrical capacitance tomography [32,33]. Wang H.X and Zhang L.F used the capacitance value that was measured by a single ECT as the input of the support vector machine (SVM) algorithm to perform GVF (gas void fraction, especially referring to sectional gas fraction) prediction with an average relative error of 10% [34].

In this paper, we propose the convolutional neural network (CNN) algorithm to realize the non-linear mapping of the flow rates and flow patterns in oil-gas two-phase flow. Two ECT sensors were used to collect the data under different flow rates. One is located in front of the venturi tube and the other is located in the end of the venturi tube. The LBP algorithm was used to image these different flow patterns. By using the information from the flow pattern diagram, the CNN algorithm is used to predict the oil flow rate, gas flow rate, and GVF. The flow pattern before venturi tube, the flow pattern after venturi tube, and the flow pattern merged before and after venturi tube are predicted, respectively. This paper improves the accuracy of measuring the flow rates of the oil-gas two- phase flow while using the typical ECT measurement technology combined with the machine learning algorithm.

## 2. Methodology

### 2.1. Electrical Capacitance Tomography (ECT)

#### 2.1.1. The Sensor

Electrical capacitance tomography consists of three main components: sensors, the system of data acquisition, and the system of computer imaging. The system of data acquisition applies voltage to each electrode plate of the sensor, and then obtains the capacitance value between any two plates by demodulating the voltage value between the excitation plate and the ground plate; the collected capacitance value is input into the computer imaging. The system performs normalization processing, and the computer uses the normalized capacitance value and image reconstruction algorithms to reconstruct the distribution of the internal medium of the measured fluid.

The commonly used ECT sensor consists of eight plates. For a sensor consisting of M electrode plates, the number of independent capacitors is M(M−1)/2 when only one electrode is energized and all the other electrodes remain at zero potential [35].

The boundary condition for ECT is that the potential distribution of the excitation electrode is ϕ=V and the potential distribution of the fixed electrode is ϕ=0. The relationship between the capacitance and dielectric constant distribution is as follows:

(1) $$C=\frac{Q}{V}=-\frac{1}{V}\iint_{\sigma }\eta (x,y)\nabla \mu (x,y)d\sigma$$

*Q* is the charge, η(x,y) is the distribution of dielectric constant, μ(x,y) is the distribution of potential in the sensing region, and σ is the unit charge on electrode surface.

#### 2.1.2. Image Reconstruction

ECT image reconstruction is an inverse problem, which is based on capacitance measurements between electrode pairs to confirm the electrical constants distribution in a pipeline. There is a nonlinear relationship between the measured capacitance value and the dielectric constant, which can be simplified as [36]:

(2) λ=Sg

λ is the normalized value of the capacitance vector, S is the normalized sensitive field matrix, and g is the distribution matrix inside the medium (normalized dielectric constant). For the ECT system, the normalization formula for λ is as follows [36]:

(3) $$\lambda_{normal}=\frac{\lambda -\lambda_{\min}}{\lambda_{\max}-\lambda_{\min}}$$

$\lambda_{\min}$ is the capacitance measurement vector when the pipe is full of gas and $\lambda_{\max}$ is the capacitance measurement vector when the pipe is full of oil. For a two-dimensional field, the sensitive field matrix S can be solved by the following formula [36]:

(4) $$S_{m,n}(q)=-\int_{\tau }\frac{\vec{E_{m}}(q)}{V_{m}}\cdot \frac{\vec{E_{n}}(q)}{V_{n}}ds,\;q\;=\;1,2,\ldots k$$

$S_{m,n}(q)$ is the sensitivity of the electrode to the qth cell of m−n, $\vec{E_{m}}(q)$ is the distribution of electric field intensity under the condition of applying voltage excitation $V_{m}$ to electrode m, and other electrodes are under grounding conditions. $\vec{E_{n}}$ is the same and τ is the area of the grid *q*. In imaging, it is often necessary to normalize the sensitive matrix, as follows:

(5) $$S_{mn}^{*}=\frac{S_{mn}}{\sum_{q=1}^{k}S_{mn}}$$

After obtaining the sensitive matrix S, the formula for calculating the medium distribution matrix can be obtained:

(6) $$g=S^{-1}\lambda$$

In most cases, the sensitive matrix S is irreversible, so $S^{-1}$ does not exist. Therefore, the linear projection algorithm (LBP) is often used for image reconstruction. The algorithm uses the transposed matrix $S^{T}$ of the sensitive field matrix S instead of the inverse matrix $S^{-1}$ of the sensitive field matrix to calculate the medium distribution matrix g [37], which is:

(7) $$g=S^{T}\lambda$$

It can be obtained by normalization:

(8) $$\hat{g}=\frac{S^{T}\lambda }{S^{T}u}$$

u is the unit vector. The algorithm has simple principle, fast imaging speed, and wide application range. In this paper, the LBP algorithm is used to obtain the flow patterns of oil-gas two-phase flow, before and after the venturi tube. The flow patterns are obtained by inputting 28 sets of capacitance values.

### 2.2. Gas Void Fraction (GVF)

#### 2.2.1. The Real GVF Calculation Formula

The calculation formula of real GVF β is shown in Equation (9).

(9) $$\beta =\frac{Q_{g}}{Q}=\frac{Q_{g}}{Q_{g}+Q_{l}}$$

In Equation (9), $Q_{g}$ represents gas volume flow, Q represents the total volume flow, and $Q_{l}$ represents liquid volume flow, which herein represents the oil volume flow rate. $Q_{g}$ and $Q_{l}$ are obtained through experimental measurements.

#### 2.2.2. Typical-ECT-Imaging-Based-GVF Algorithm 

We set the threshold to process the flow pattern diagram according to the pixel value of the reconstructed flow pattern diagram. The threshold is set to 0.65, setting the pixel above the threshold (i.e., oil) as 1, and the pixel below the threshold (i.e., air) as 0, the ratio of the number of pixels of air to the total number of pixels is the gas void fraction (GVF, especially referring to sectional gas fraction) in the current state. According to the pixel value of the flow pattern diagram, the calculation formula of gas void fraction (GVF) is shown in Equation (10).

(10) $$GVF=(1-\sum_{j=1}^{M}f_{j}\frac{A_{j}}{A})\times 100\%$$

M is the total number of pixels in the section, $f_{j}$ is the gray value of the $j^{th}$ pixel, $A_{j}$ is the area of the $j^{th}$ pixel, and A is the total area of the pipeline section.

### 2.3. SVM (Support Vector Machine) Algorithm

This paper uses the regression model of support vector machine, while using the Function (11):

(11) f(x)=<ω,ϕ(x)>+b.

Subsequently, it can derive the Equation (12):

(12) f(y)=<ω,ϕ(y)>+b.

x is an independent variable, y is a dependent variable, ω is a weight vector, b is an offset, and $\phi (x):R^{d}\to H$ is a nonlinear function that maps the data set *S* to a high-dimensional linear eigenspace and seeks optimality in the eigenvector. For the regression function, the optimization goals and constraints of the SVM are Equations (13) and (14), respectively. The ε insensitive loss function is used for a given training data set. The corresponding support vector machine is the so-called ε-Support Vector Machine [38].

(13) $$\underset{\sigma }{\min}\frac{1}{2}||\sigma ||+K\sum_{j=1}^{M}(\eta_{j}+\eta_{j}^{*}),\;\;\;j=1,2,\ldots m$$

(14) $$s.t.\{\begin{array}{c} y_{j}-\sigma \cdot \phi (x)-b\leq \varepsilon +\eta_{j}^{*}\\ \sigma \cdot \phi (x)+b-y_{j}\leq \varepsilon +\eta_{j}\\ \eta_{j},\eta_{j}^{*}\geq 0 \end{array}$$

K is the penalty coefficient, the larger value indicates the higher requirement for error, by introducing Lagrangian function, the optimization problem of Equations (13) and (14) is transformed into dual problem, which is obtained by solving the dual problem (the solution of Equation (11) [38]:

(15) $$f(x)=\langle \omega ,\phi (x)\rangle +b=\sum_{j=1}^{M}a_{j}Q(x_{j},x_{i})+b.$$

The Gaussian core is used in this paper [38]:

(16) $$Q(x_{j},x_{i})=\exp(\frac{||x_{j}-x_{i}|{|}^{2}}{\rho^{2}}).$$

$a_{j}$ is the vector difference of the Lagrangian multiplier, *b* is the constant term, which is the offset constant, and the ρ parameter is the kernel width. It can be seen from Equations (11)–(16) that the promotion ability of SVM can be controlled by controlling K, ε, and ρ.

In this paper, the independent variable *x* is the flow pattern that is obtained by the LBP algorithm, and the dependent variable *y* is the oil flow rate, gas flow rate, and GVF corresponding to the flow patterns. Finally, the optimal regression function between the image high-dimensional data and the feature vectors is obtained [38]. 

### 2.4. Convolutional Neural Network (CNN)

**Figure 1:**
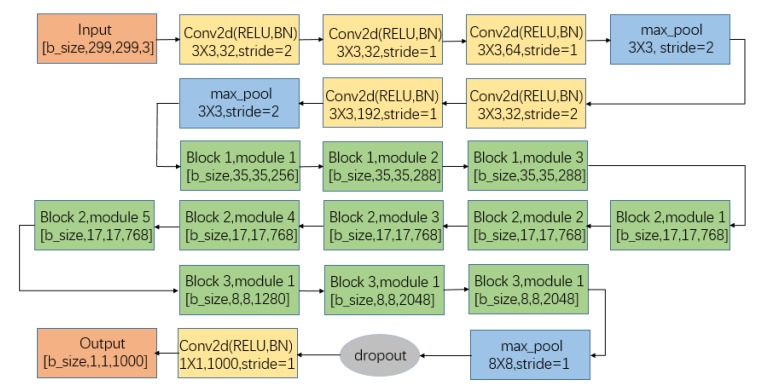
Convolutional neural network (CNN) network model structure diagram.

The image reconstruction algorithm can obtain the flow patterns under different working conditions. In this paper, the CNN algorithm is used to solve the nonlinear mapping of oil-gas two-phase flow parameters (GVF and flow rates) to the change of flow patterns. The input of the CNN model is the flow patterns before and after the venturi tube, and the output is the oil flow rate, gas flow rate, and GVF under the corresponding state of the flow patterns. The traditional regression algorithm cannot solve the relationship between high-dimensional data (streaming image pixels) and low-dimensional data (GVF and flow rates). In the CNN algorithm, increasing the nonlinear activation response can decouple more nonlinear characteristics, thus the training speed of the network can be improved [39]. In this paper, the Inception V3 model of the CNN algorithm is used to solve the nonlinear mapping of oil-gas two-phase flow parameters (GVF and flow rates) and the changes of flow patterns before and after the venturi tube. Figure 1 shows the model structure diagram [39].

As an extremely deep CNN model, Inception V3 has a very sophisticated design and construction. The structure and branches of the entire network are very complicated. The convolutional network is gradually reduced in size from input to output, and the number of output channels is gradually increased. The spatial information is transformed into high order abstract information by simplifying the spatial structure. Factorization into small convolutions is very effective, it can reduce the amount of parameters, reduce over-fitting, and enhance the nonlinear expression of the network [39]. It is very suitable for solving the high-dimensional and nonlinear relationship between the flow patterns and the GVF. In this paper, the input layer of the neural network is flow patterns that were obtained by image reconstruction, and the output layer has two neurons for outputting the predicted values of gas and oil flow rates.

When compared to the fully connected neural network, the CNN algorithm implements local connectivity, weight sharing, and down-sampling. For the input image, this algorithm can achieve better learning effects by retaining important parameters as much as possible and removing a large number of unimportant parameters [39].

The forward propagation algorithm in neural networks can be expressed as [39]:

(17) $$k_{m}^{h}=f(\sum_{n=1}^{S^{h-1}}w_{mn}{}^{h}k_{n}{}^{h-1}+q_{m}{}^{h})$$

$k_{m}^{h}$ is the result of the $m^{th}$ neuron in the $h^{th}$ neural network, $k_{n}{}^{h-1}$ is the result of the $n^{th}$ neuron in the ${\left(h-1\right)}^{th}$ neural network, and $w_{mn}{}^{h}$ is the $h^{th}$ neuron of the ${\left(h-1\right)}^{th}$ layer to the $h^{th}$ layer. $q_{m}{}^{h}$ is the deviation of the $m^{th}$ neuron in the $h^{th}$ layer neural network. The Relu activation function that is used in this paper is expressed, as follows [39]:

(18) f(x)=max(x,0)

The loss function of the network uses ElasticNet regression, which is a mixture of Ridge and Lasso regression techniques. In the case of highly correlated variables, the group effect is generated. The number of selected variables is not limited and it can withstand double contraction. When compared to the least squares regression, ElasticNet regression effectively avoids possible over-fitting problems. The input of the network in this paper is an image, which belongs to high-dimensional data. ElasticNet regression has obvious effects in the case of multi-collinearity between high-dimensional and data-set variables. Its objective function is shown in Equation (18) [39]:

(19) $$e=\frac{1}{n}[\sum_{i=1}^{n}(h_{\omega }(x^{\left(i\right)})-y^{\left(i\right)}{)}^{2}+\lambda_{1}||\omega |{|}_{1}+\lambda_{2}||\omega |{|}^{2}]=T(\omega )$$

e is the error between the true and predicted value, $h_{\omega }(x^{\left(i\right)})$ is the predicted oil flow rate and gas flow rate, $y^{\left(i\right)}$ is the true oil flow rate and gas flow rate, *n* is the number of data sets, and $\lambda_{1}$ and $\lambda_{2}$ are regularization parameters. ω is a vector containing weights and deviations between individual neurons [39].

By using the back propagation of the CNN to update the weights, the training model uses the Inception V3 network in the CNN. In this paper, the ECT data is first measured by a large number of experiments, and the acquired ECT data re imaged by a linear projection algorithm to obtain the flow patterns. Under different working conditions, the flow patterns before and after the venturi tube are input the CNN model to predict the oil flow rate, gas flow rate, and GVF.

For the CNN algorithm, the real-time parameters of the algorithm are frames per second (FPS), and the complexity parameters of the algorithm are params and floating-point operations per second (FLOPS) of the model. Algorithm real-time FPS that is based on Nvidia 1080Ti graphics card (NVIDIA, Santa Clara, CA, USA) measured FPS is 130.2, its params is 27.16 (M), and FLOPS is 5.75 (G).

## 3. Experiment

The experiment was completed on a semi-industrial multiphase flow experimental measuring platform. The overall experimental equipment is shown below. Oil is stored in a separator, being separated according to the principle of gravity and a gas phase compressor produces the gas. Natural gas and oil flow through single-phase pipelines, the mixture of oil and gas can pass through the test pipe. The test pipe is eight meters long, and the ECT sensor and venturi tube are on the test pipe. The maximum pressure of the device is 2 MPa. The detailed dimensional layout of the venturi tube and ECT sensor is given below. The length of the venturi tube is 1200 mm, the length of the inner diameter is 50 mm, the length of the outer diameter is 60 mm, the length of the throat tube is 130 mm, and the opening Angle is 18°. The ECT sensor has an internal structure of eight electrodes, and the insulation wall of ECT sensor is 5 mm thick. Its length is 60 mm. The length of the internal diameter is 50 mm. The length of the external diameter is 86 mm and the internal electrode size is 192 mm × 90 mm.

**Figure 2:**
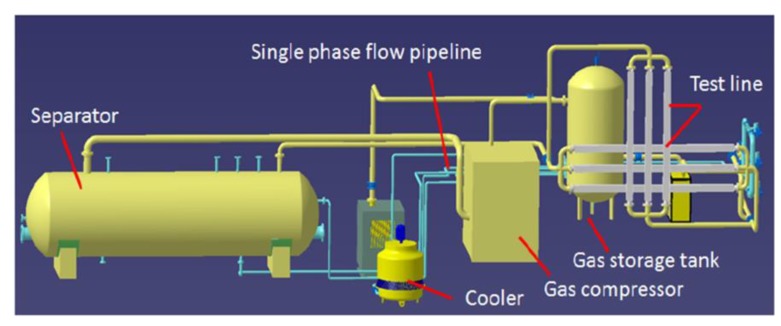
Schematic diagram of experimental equipment.

Figure 2 shows the schematic diagram of the experimental equipment used to perform this experiment. In this experiment, there are two ECT sensors that are located on upstream and downstream of the venturi tube. There are 8 electrodes inside each ECT sensor. The acquisition frequency of ECT system is 100 frames/s, and the excitation signal frequency of ECT is 100 kHz. The SNR (signal to noise ratio) of the hardware system is about 62 dB. 

The experimental material is No.15 industrial white oil, having a relative dielectric constant of 2.2, the density is 880 kg/m^3^, and the viscosity is 8.8 mPa s (33 °C), white oil contains high levels of cycloparaffin (MOSH) and 25% Alkyl substituted aromatic hydrocarbons (MOAH). The gas is natural gas, which is consistent with the oilfield site environment. The working pressure is 0.6 MPa. The experimental temperature is 33 °C while using the temperature transmitter.

In this experiment, we collected a large amount of experimental data to train the model. Through the semi-industrial multiphase flow experimental measurement platform, the data collected time under each working condition is 10 min, and the collected data are the capacitance value of ECT sensor. The LBP image reconstruction algorithm is used to convert the capacitance into flow pattern for model training. The train set was collected under 52 working conditions. There were 8000 training samples in each working condition, and the total number of training samples was 416,000. The test set was also collected under the same 52 working conditions. There were 2000 test samples in each working condition, and the total number of test samples was 104,000. Table 1 shows the specific oil flow rate, gas flow rate, and GVF of the 52 working conditions in the experiment.

**Figure 3:**
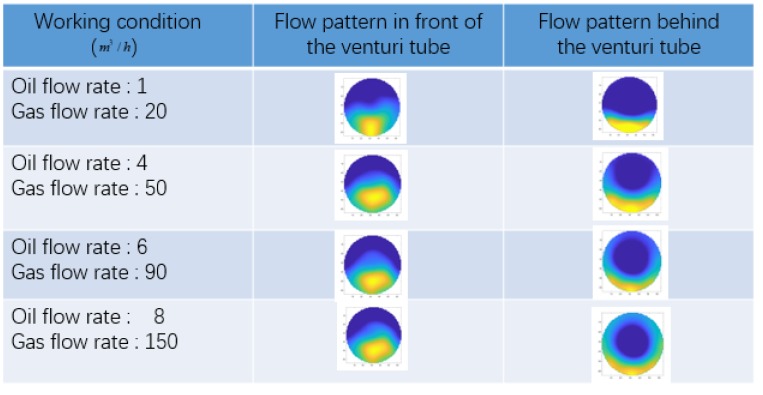
Averaged electrical capacitance tomography (ECT) image (100 frames) of flow distribution before and after venturi tube.

Table 1 shows the oil flow rate and gas flow rate distribution of 52 working conditions. In the actual experiment, when collecting experimental data, the oil flow rate and gas flow rate of each working condition are not a fixed value. It will randomly fluctuate by 15% above or below the set working condition. Accordingly, the data of the training set and the test set are not same, which effectively verifies the generality of the trained model. The flow pattern diagram that is shown in Figure 3 is the representative of the flow pattern diagram under some typical working conditions. Figure 3 shows the flow patterns change before and after the venturi tube. The values of GVF in the experiment are evenly distributed between 0.25 and 0.95 in order to ensure the full coverage of GVF. The data collection began once the single flow rate was stable, and the oil and gas mixing was completed.

## 4. Analysis and Discussion

### 4.1. Flow Data Analysis

In this experiment, the two-phase flow flows through the venturi tube, ECT data before and after the venturi tube are collected. The LBP algorithm is used to image the flow distributions. The ECT images vary a lot due to the noise in measurement data. The average ECT images were averaged by 100 pieces of ECT data, and the flow patterns tend to be stable and they are convenient for analysis. The following is the flow patterns before and after the venturi tube under typical working conditions. Figure 3 shows the flow patterns before and after the venturi tube under typical working conditions.

**Figure 4:**
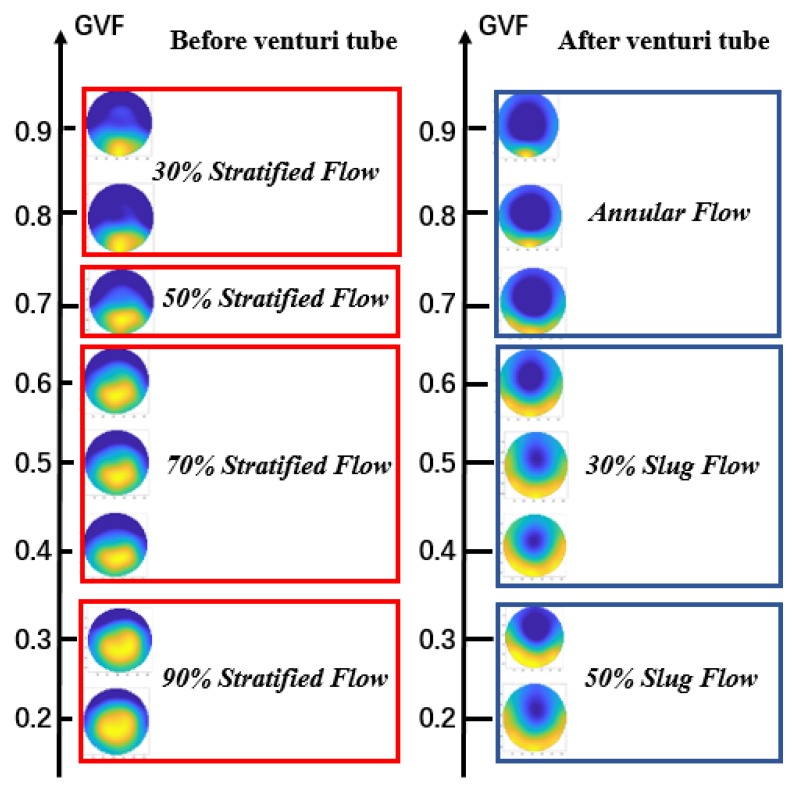
Flow pattern diagram under typical gas void fraction (GVF).

Figure 3 shows the flow pattern under typical working conditions. It can be seen from the Figure 3 that, under, different oil and gas flow rate, the flow pattern in front of the venturi tube changes little, but after the oil-gas two-phase flow passes through the venturi tube, the flow pattern changes greatly. When the gas flow rate is small, the flow pattern is stratified flow. With the increase of gas flow rate, the flow pattern becomes slug flow. The flow pattern becomes annular flow when the gas flow rate continues to increase. Figure 4 shows the flow patterns change before and after the venturi tube under typical GVF.

From Figure 4, it can be seen that flow patterns change before and after the venturi tube when the GVF value changes from 0.2 to 0.9. When the GVF is less than 0.6, the flow patterns before the venturi tube is 90% stratified flow and 70% stratified flow, while the flow patterns after the venturi tube is slug flow. When the GVF is more than 0.6, the flow pattern diagram before the venturi tube is still stratified flow, but the flow pattern diagram after the venturi tube becomes annular flow. When the oil flow rate is more than 5 m^3^/h, the annular flow trend of the flow patterns after the venturi tube is more obvious. Based on this phenomenon, the CNN network is used to predict the oil flow rates and gas flow rates while using the ECT images as the input. 

### 4.2. Flow Pattern Recognition and Prediction

In this paper, the flow patterns in front of the venturi tube, the flow patterns after the venturi tube, and the flow patterns merged before and after the venturi tube are respectively input into the network for prediction, and the relative error is compared. This paper uses the Inception V3 model in CNN to predict the flow rates under different conditions since the relationship between flow patterns and oil and gas flow rate is non-linear. The flow patterns in front of the venturi tube, the flow patterns after the venturi tube, and the flow patterns merged before and after the venturi tube were respectively trained to compare the predicted performance of oil flow rate, gas flow rates, and GVF. 

Firstly, the flow patterns were obtained according to the LBP image reconstruction algorithm, and the corresponding GVF (gas void fraction) in the current flow patterns was calculated while using the Typical-ECT-image-based-GVF algorithm. We set the threshold to process the oil-gas two-phase flow imaging image. The threshold is set to 0.65, setting the pixel above the threshold (i.e., oil) as 1, and the pixel below the threshold (i.e., air) as 0. The ratio of the number of pixels of air to the total number of pixels is the gas void fraction (GVF) in the current state. 

**Figure 5:**
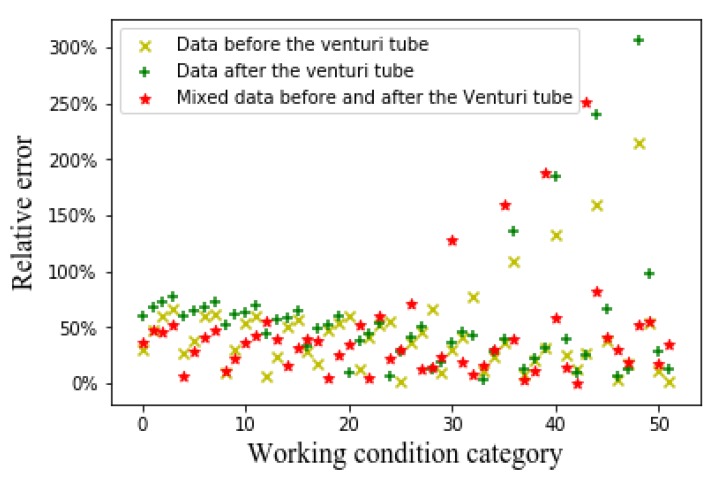
Relative error of GVF obtained from ECT images.

We use the Typical-ECT-image-based-GVF algorithm to predict the GVF and obtain the average relative error of the prediction. Figure 5 shows the relative error of predicted GVF from the ECT images. 

From Figure 5 (the *X*-axis represents 52 working conditions, i.e., the range of oil flow rate is 1–10 m^3^/h, the range of gas flow rate is 20–150 m^3^/h, and the range of GVF is 0.25–0.95. The *Y*-axis represents the average relative error of each case) it can be seen that whether it is the flow patterns before the venturi tube, the flow patterns after the venturi tube, and the flow patterns before and after the venturi tube. The effect of Typical-ECT-image-based-GVF algorithm for predicting GVF is poor. This might be due to the low accuracy of the LBP algorithm. The LBP algorithm has better real-time performance, but the imaging accuracy is worse than that of various complex iterative algorithms. It affects the accuracy of the GVF measurements to some extent.

Meanwhile, the SVM algorithm is a commonly used algorithm for GVF prediction of oil-gas two-phase flow. The flow patterns that are obtained by linear projection algorithm (LBP) are input into the model, and the oil flow rate and gas flow rate corresponding to each flow pattern are respectively input, to find out the non-linear relationship between the flow patterns and oil flow rate, gas flow rate, and GVF. The oil flow rate and gas flow rates predicted from 10,000 pictures under each working condition were averaged, respectively. We compare the actual value of the oil flow rates and gas flow rates with the predicted value.

**Figure 6:**
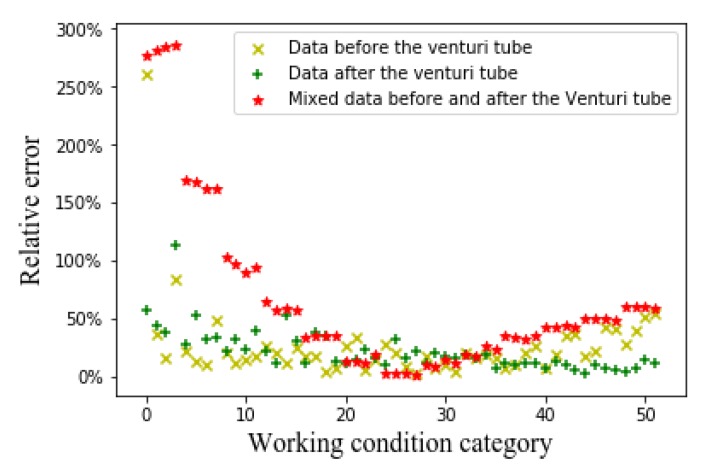
Relative error of oil flow rate with support vector machine (SVM) algorithm.

**Figure 7:**
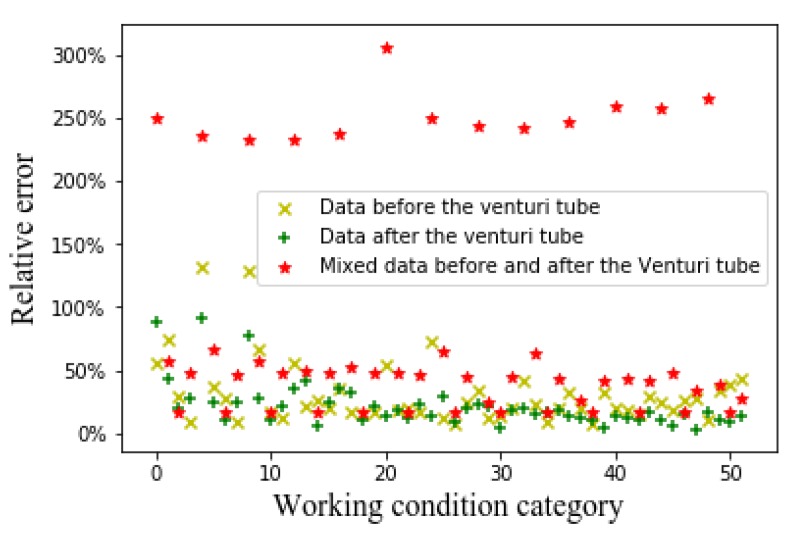
Relative error of gas flow rate with the SVM algorithm.

**Figure 8:**
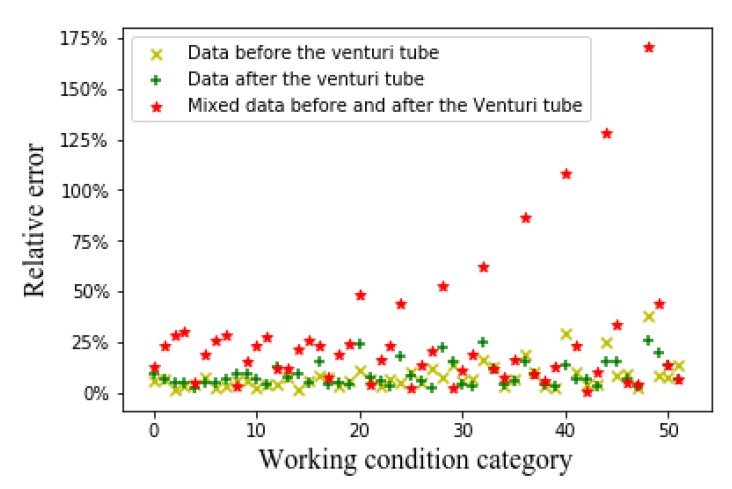
Relative error of GVF with SVM algorithm.

Figure 6, Figure 7 and Figure 8, respectively, show the relative errors in predicting the oil flow rate, gas flow rate, and GVF of oil-gas two-phase flows while using the SVM algorithm. For the prediction of oil flow rate, whether it is the flow patterns in front of the venturi tube, the flow pattern after the venturi tube, or the flow patterns before and after the venturi tube, the relative error is large at low flow rates, as can be seen from Figure 6. As the oil flow rate increases, the predicted relative error decreases. As can be seen from Figure 7 and Figure 8, for the gas flow rate and GVF prediction, in the small gas flow rate, the relative error is large. This shows that the SVM algorithm is only suitable for the prediction of large oil and gas flow rate, which might be related to the limitations of the SVM algorithm itself. The principle of SVM algorithm is based on existing data, and the hyperplane is fitted to predict, which has certain limitations on high-dimensional data. The CNN algorithm is introduced to solve the relationship between high-dimensional data (flow pattern diagram pixels) and low-dimensional data (GVF and flow rates) to solve the problem.

The training model uses the Inception V3 network in a CNN algorithm. Since the input is image pixel data, the data dimension is high and the amount of data is large. With the Inception V3 network, large two-dimensional (2D) volumes can be integrated into two smaller convolutions. In the CNN model, there are 416,000 samples in the training set and 104,000 samples in the test set. The oil flow rates, gas flow rates, and GVF predicted by 10,000 images under each condition were averaged and then compared with the actual values. The oil flow rate, gas flow rate, and GVF under 52 conditions were predicted and the relative error was obtained.

**Figure 9:**
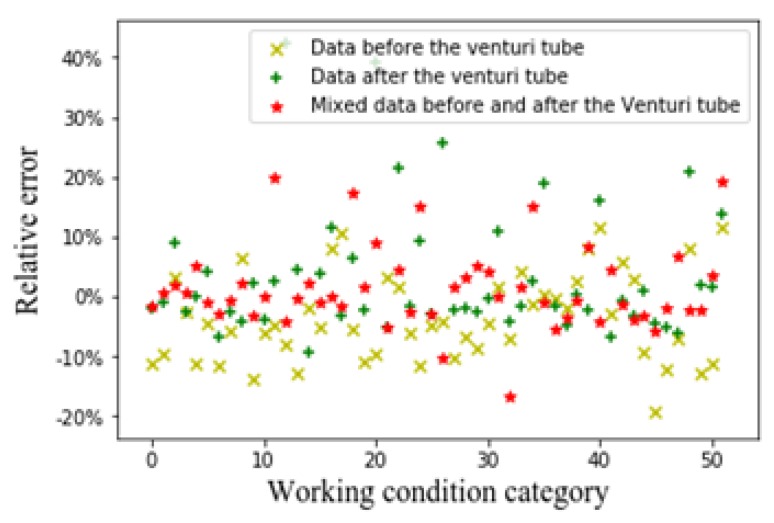
Relative error of oil flow rate with CNN algorithm.

**Figure 10:**
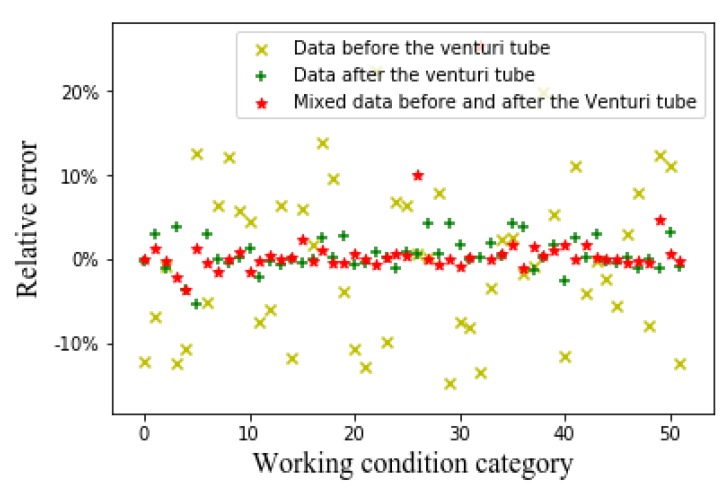
Relative error of gas flow rate with CNN algorithm.

**Figure 11:**
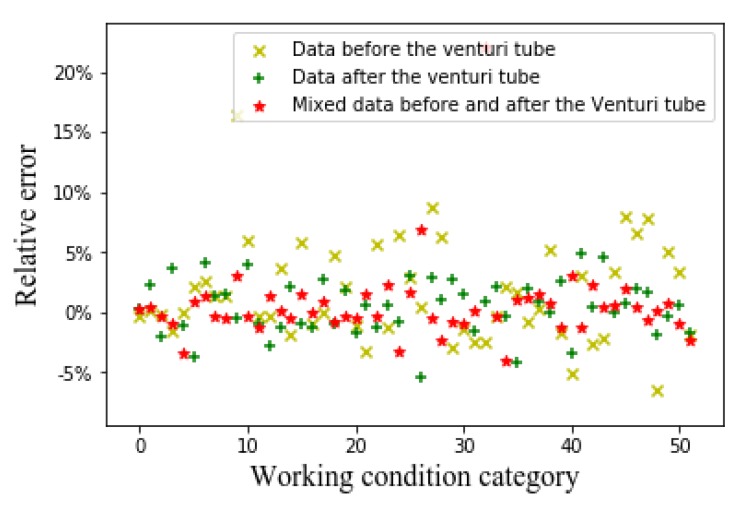
Relative error of GVF with CNN algorithm.

Figure 9, Figure 10 and Figure 11, respectively, show the relative errors in predicting the oil flow rate, gas flow rate, and GVF of oil-gas two-phase flows while using the CNN algorithm. It can be seen from Figure 9 that using CNN algorithm to predict the oil flow rate, the prediction results of the merged flow patterns before and after the venturi tube are superior to those prediction results of the flow patterns before the venturi tube and the flow patterns after the venturi tube. It can be seen from Figure 10 and Figure 11 that, while using the CNN algorithm to predict the gas flow rate and GVF, the prediction relative errors are less than 5% through the merged flow patterns before and after the venturi tube.

As can be found, the prediction accuracy of the CNN algorithm has been significantly improved when compared with the Typical-ECT-imaging-based-GVF algorithm and SVM algorithm. It can be seen that the CNN network is suitable for the regression of high-dimensional image data and greatly improved the predicted results. 

The flow patterns after the venturi tube, and the merged flow patterns before and after the venturi tube, the average prediction relative error of the three sets of flow patterns is analyzed below to compare the flow patterns before the venturi tube.

From Table 2, Table 3 and Table 4, CNN algorithm can provide the most accurate prediction of gas flow rates and oil flow rates. Meanwhile, using the ECT data that were collected before and after the venturi tube can also improve the accuracy of the measurement of the flow rates. 

Among them, while using the CNN algorithm, the average relative error of oil flow rate prediction is 4.6%, the predicted relative error of gas flow rate is 1.4%, and the average relative error of GVF prediction is 1.6%. 

In terms of the measurement of GVF by using the ECT sensor before the venturi tube with SVM algorithm, 90 percent results of 104,000 test samples are less than the relative error with 13.8%, and with CNN algorithm, 90 percent results are less than the relative error with 4.48%, it can be seen that the CNN algorithm is better than the SVM algorithm. When compared to the data before the venturi tube, the measurement of GVF by using the data after the venturi tube with CNN algorithm, 90 percent relative error of 104,000 test samples are less than 3.74%, and it can be seen that the prediction effect of the data after the venturi tube is better. 90 percent relative error of 104,000 test samples are less than 3.06% by using the mixed data before and after the venturi tube with CNN algorithm. It can be seen that the prediction effect of the merged flow patterns before and after the venturi tube is the best.

Finally, for oil flow rate prediction, the relative error of 90% is less than 11%; for gas flow rate prediction, the relative error of 95% is less than 3.6%, and that of 90% is less than 2.2%; and, for GVF prediction, the relative error of 95% is less than 3.3% and that of 90% is less than 3%.

### 4.3. Raw Capacitance Data (Comparative Experiment)

**Figure 12:**
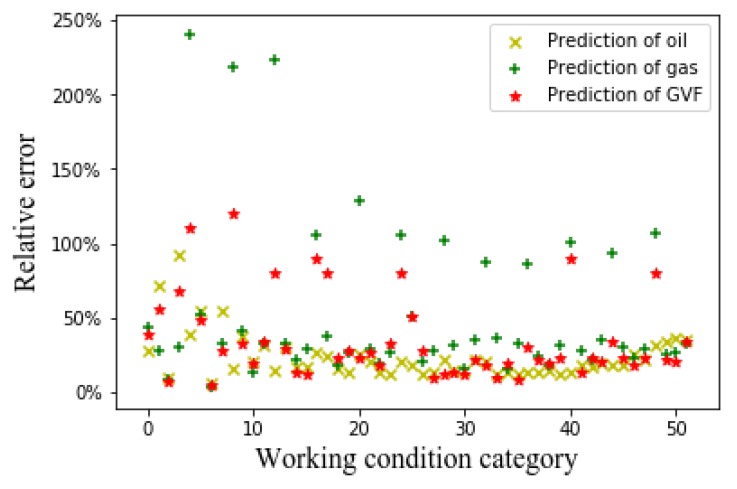
Relative error of SVM algorithm based on original capacitance value.

We use the original capacitance data as input to perform the flow prediction in order to compare with the experimental prediction results of the input of the flow pattern. The SVM algorithm and CNN algorithm were used to predict the oil content, gas content, and GVF, respectively. The eight-electrode ECT sensor generates 28 sets of capacitance values. First, the 28 sets of capacitance values are input to the SVM algorithm for flow prediction. The training data set is 8000 and the test data set is 2000. In the traditional SVM regression algorithm, the capacitance value needs to be normalized in order to compare with the experimental prediction results of the input of the flow pattern (if the normalization is not performed, the algorithm cannot converge). The prediction results are shown in Figure 12.

It can be seen from Figure 12 that the prediction result of the SVM algorithm that is based on the original capacitance value is significantly worse than the prediction result of the CNN algorithm based on the flow pattern diagram. The average relative error of the SVM algorithm that is based on the original capacitance value is 0.47. The SVM algorithm is sensitive to the parameter adjustment and the choice of the kernel function, and it has a poor effect on the regression problem. 

**Figure 13:**
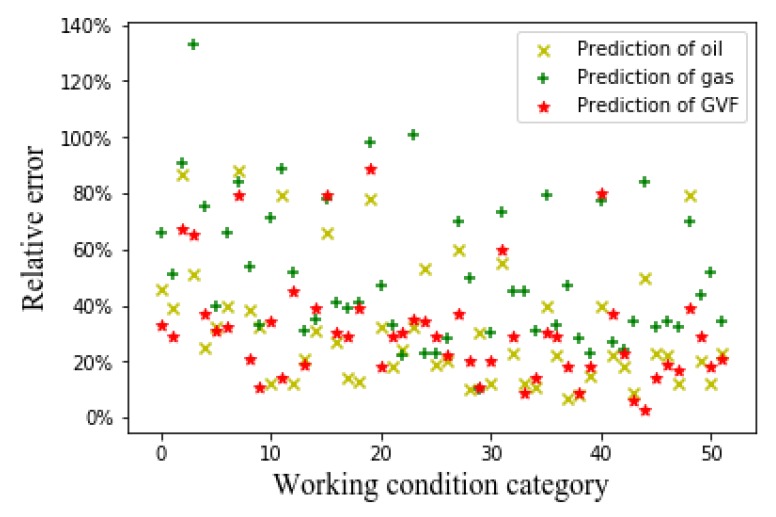
Relative error of CNN algorithm based on original capacitance value.

Furthermore, we use the CNN algorithm with the original capacitance value as the input to perform flow rate prediction. The training set has a total of 416,000 and the test set has a total of 104,000. Figure 13 shows the flow rate prediction result.

When comparing with the prediction results of the CNN algorithm based on the flow patterns, the prediction result of the CNN algorithm based on 28 original capacitance values is obviously poor, as can be seen from Figure 13. The average relative error of the CNN algorithm based on 28 original capacitance values is: 0.33. It can be seen that modeling and analysis that are based on the flow pattern diagram have better prediction results.

The CNN algorithm is suitable for information extraction and the fitting of two-dimensional data because of its application of a large number of convolution operations. For 28 capacitance values in one dimension, the CNN algorithm has a very poor extraction effect on its effective information. Therefore, this paper reconstructs the flow pattern diagram that is based on 28 capacitance values, which makes the CNN algorithm extract features more efficiently. From a mathematical perspective, the purpose of this algorithm to reconstruct 28 capacitance values into a flow pattern is to make its representation more suitable for CNN algorithm to extract features.

The above is based on the analysis of the capacitance data of the dual ECT sensors before and after the venturi tube, and the prediction results for the capacitance data of a single ECT sensor are similar to those that are shown in Figure 13.

It can be seen that the prediction effect of using the flow pattern image as the input of the CNN model is better than that of the original capacitance value input to the CNN model. For the input of the flow pattern image, it can be seen that, whether it is the comparison of the average relative error or the comparison of the relative error of 90% measuring results, the best prediction effect can be achieved by predicting the merged flow patterns before and after the venturi tube by the CNN algorithm. This shows that the predictive accuracy of oil flow rate, gas flow rate, and GVF with the dual ECT sensors is better than the single ECT. The CNN algorithm is used to solve the relationship between high-dimensional data (pixels of the flow pattern diagram) and low-dimensional data (GVF and flow rate) that cannot be solved by traditional algorithms. The prediction effect is better when compared with traditional prediction algorithms.

## 5. Conclusions

It can be seen from the experiment that the prediction result of using the flow pattern image as the input of the CNN algorithm is much better than the prediction result of the input of the original capacitance value. Through the experiment, it can be seen that the flow patterns of oil-gas two-phase flow before and after venturi tube is different, with the different flow rates, the varying behavior will be different. The oil flow rate, gas flow rate, and GVF under current working conditions are predicted by the flow patterns change before and after the venturi tube. While considering the nonlinear relationship between flow pattern diagrams with flow rates, a convolutional neural network algorithm is proposed to predict gas and oil flow rate. The CNN model has accurate and stable performance. The experimental results show that the improved CNN model has higher prediction performance, the average relative error of oil flow rate is 4.6%; the average relative error of gas flow rate is 1.4%; and, the average relative error of GVF is 1.6%.

In summary, the CNN algorithm greatly improves the predictive accuracy of GVF. It is of great significance to accurately measure the parameters of oil-gas two-phase flow. Unfortunately, the CNN algorithm requires high computational force; at the present stage, it is unable to realize the rapid deployment of measurement devices in the industry, and it is also unable to convert them into portable devices. In addition, the ECT image reconstruction algorithm should be further improved to make its computational efficiency more in line with the needs of practical industrial applications. Therefore, this method is not easy to realize in real-time flow measurement in industrial field. In the future, we will conduct in depth research on the lightweight and embedded model. 

## Figures and Tables

**Table 1:** Working condition distribution table m^3^/h.

Oil Gas	1	1.5	2	2.5	3	3.5	4	4.5	5	6	7	8	10
20	1	5	∙ ∙ ∙									$\begin{array}{l} \cdot \\ \cdot \\ \cdot \end{array}$	49
50	2	6											50
90	3	$\begin{array}{l} \cdot \\ \cdot \\ \cdot \end{array}$										47	51
150	4											48	52

**Table 2:** Average relative error table of prediction of data before the venturi tube.

	Binarized Image	SVM	CNN
Oil flow rate	no	23.01%	7.02%
Gas flow rate	no	30.66%	7.12%
GVF	43.85%	8.23%	5.01%

**Table 3:** Average relative error table of prediction of data after the venturi tube.

	Binarized Image	SVM	CNN
Oil flow rate	no	22.18%	7.03%
Gas flow ate	no	21.59%	1.47%
GVF	56.30%	9.13%	1.88%

**Table 4:** Average relative error table of prediction of Mixed data before and after the venturi tube.

	Binarized Image	SVM	CNN
Oil flow rate	no	67.12%	4.66%
Gas flow rate	no	89.88%	1.43%
GVF	43.09%	27.04%	1.67%

## References

[1] Omar R., Hewakandamby B., Azzi A., Azzopardi B. (2018). Fluid structure behaviour in gas-oil two-phase flow in a moderately large diameter vertical pipe. Chem. Eng. Sci..

[2] Hanafizadeh P., Eshraghi J., Nazari Y., Yousefpour K., Behabadi M.A.A. (2017). Light oil—Gas two-phase flow pattern identification in diffesrent pipe orientations: An experimental approach. Sci. Iran. Trans. B Mech. Eng..

[3] Drury R., Hunt A., Brusey J. (2019). Identification of horizontal slug flow structures for application in selective cross-correlation metering. Flow Meas. Instrum..

[4] Mohmmed A.O., Nasif M.S., Al-Kayiem H.H., Time R.W. (2016). Measurements of translational slug velocity and slug length using an image processing technique Flow. Meas. Instrum..

[5] Abbagoni B.M., Yeung H. (2016). Non-invasive classification of gas-liquid two-phase horizontal flow regimes using an ultrasonic Doppler sensor and a neural network. Meas. Sci. Technol..

[6] Dong X., Tan C., Yuan Y., Dong F. (2016). Measuring oil-water two-phase flow velocity with continuous-wave ultrasound doppler sensor and drift-flux model. IEEE Trans. Instrum. Meas..

[7] Aarabi Jeshvaghani P., Feghhi S.A.H., Khorsandi M. (2019). Temperature independent flow-rate prediction in two-phase flow loop using gamma-ray attenuation and Artificial Neural Networks. Radiat. Meas..

[8] Lupeau A., Platet B., Gajan P., Strzelecki A., Escande J., Couput J.P. (2007). Influence of the presence of an upstream annular liquid film on the wet gas flow measured by a Venturi in a downward vertical configuration. Flow Meas. Instrum..

[9] Steven R. (2008). A dimensional analysis of two phase flow through a horizontally installed Venturi flow meter. Flow Meas. Instrum..

[10] Meng Z.Z., Huang Z., Wang B., Ji H., Li H., Yan Y. (2010). Air—Water two-phase flow measurement using a Venturi meter and an electrical resistance tomography sensor. Flow Meas. Instrum..

[11] Alssayh M.A., Addali A., Mba D. Determining slug velocityin two-phase flow with acoustic emission. Proceedings of the 51st Annual Conference of the British Institute of Non-Destructive Testing, NDT.

[12] Liu L., Bai B. (2019). Flow regime identification of swirling gas-liquid flow with image processing technique and neural networks. Chem. Eng. Sci..

[13] Chen S.W., Brooks C.S., Macke C., Hibiki T., Ishii M., Mori M. Experiment of adiabatic two-phase flow in an annulus under low-frequency vibration. Proceedings of the 20th International Conference of Nuclear Engineering (ICONE20).

[14] Parham P. Pattern recognition in real time using neural networks: An application for pressure measurement. Proceedings of the ICPRAM 2016—5th International Conference on Pattern Recognition Applications and Methods.

[15] Kesana N.R., Parsi M., Vieira R.E., Azzopardi B., Schleicher E., McLaury B.S., Shirazi S.A. (2017). Uwe Hampel Visualization of gas-liquid multiphase pseudo-slug flow using Wire-Mesh Sensor. J. Nat. Gas. Sci. Eng..

[16] Fasching G.E., Smith N.S. (1988). High Resolution Capacitance Imaging System.

[17] Halow J.S., Nicoletti P. (1992). Observations of fluidized bed coalescence using capacitance imaging. Powder Technol..

[18] Ismail I., Gamio J.C., Bukhari S.F.A., Yang W.Q. (2005). Tomography for multi-phase flow measurement in the oil industry. Flow Meas. Instrum..

[19] Zhang L.F., Wang H.X. Identification of two-phase flow regime based on electrical capacitance tomography and soft-sensing technique. Proceedings of the 7th International Symposium on Instrumentation and Control Technology OCT.

[20] Guo Q., Ye M., Yang W.Q., Liu Z.M. (2019). A machine learning approach for electrical capacitance tomography measurement of gas-solid fluidized beds. AIChE J..

[21] Bertoldi D., Dallalba C.C.S., Barbosa J.R. (2015). Experimental investigation of two-phase flashing flows of a binary mixture of infinite relative volatility in a Venturi tube. Exp. Therm. Fluid Sci..

[22] Luo W., Li Y., Wang Q., Li J., Liao R., Liu Z., Qinghua W. (2016). Experimental Study of Gas-Liquid Two-Phase Flow for High Velocity in Inclined Medium Size Tube and Verification of Pressure Calculation Methods. Int. J. Heat Technol..

[23] Scheaua F.D. (2016). Theoretical approaches regarding the VENTURI effect. Hidraulica.

[24] Borsuk G., Dobrowolski B., Wydrych J. (2017). A numerical analysis of metrological properties of venturi tube in the air-coal particle mixture flow measurement in power industry. E3S Web of Conferences.

[25] Kashid M.N., Kowaliński W., Renken A., Baldyga J., Kiwi-Minsker L. (2012). Analytical method to predict two-phase flow pattern in horizontal micro-capillaries. Chem. Eng. Sci..

[26] Thome J.R., El Hajal J. Two-Phase Flow Pattern Map for Evaporation in Horizontal Tubes: Latest Version. Proceedings of the 1st International Conference on Heat Transfer, Fluid Mechanics and Thermodynamics.

[27] Kosterin S.I. (1949). An Investigation of the Influence of the Diameter and Inclination of a Tube on the Hydraulic Resistance and Flow Structure of Gas-liquid Mixtures. Izvest. Akad. Nauk. SSSR Otdel Tekh Nauk..

[28] Barnea D., Luninski Y., Taitel Y. (1983). Flow pattern in horizontal and vertical two phase flow in small diameter pipes. Can. J. Chem. Eng..

[29] Caetano E.F., Shoham O., Brill J.P. (1992). Upward Vertical Two-Phase Flow Through an Annulus—Part I: Single-Phase Friction Factor, Taylor Bubble Rise Velocity, and Flow Pattern Prediction. ASME J. Energy Resour. Technol..

[30] Rosa E.S., Salgado R.M., Ohishi T., Mastelari N. (2010). Performance comparison of artificial neural networks and expert systems applied to flow pattern identification in vertical ascendant gas–liquid flows. Int. J. Multiph. Flow.

[31] Marashdeh Q., Warsito W., Fan L.-S., Teixeira F.L. (2006). Nonlinear forward problem solution for electrical capacitance tomography using feed-forward neural network. IEEE Sens. J..

[32] Marashdeh Q., Warsito W., Fan L.-S., Teixeira F.L. (2006). A nonlinear image reconstruction technique for ECT using a combined neural network approach. Meas. Sci. Technol..

[33] Xie C.G., Huang S., Beck M., Hoyle B., Thorn R., Lenn C., Snowden D. (1992). Electrical capacitance tomography for flow imaging: System model for development of image reconstruction algorithms and design of primary sensors. IEE Proc. G.

[34] Wang H.X., Zhang L.F. (2009). Identification of two-phase flow regimes based on support vector machine and electrical capacitance tomography. Meas. Sci. Technol..

[35] Liu S., Fuand L., Yang W.Q. (1999). Optimization of an iterative image reconstruction algorithm for electrical capacitance tomography. Meas. Sci. Technol..

[36] Guo Q., Meng S., Wang D., Zhao Y., Ye M., Yang W., Liu Z. (2018). Investigation of gas-solid bubbling fluidized beds using ECT with a modified Tikhonov regularization technique. AIChE J..

[37] Yang W.Q., Peng L. (2012). Image reconstruction algorithms for electrical capacitance tomography. Meas. Sci. Technol..

[38] Cortes C., Vapnik V. (1995). Support vector networks. Mach. Learn..

[39] Xia X.L., Xu C., Nan B. Inception-v3 for Flower Classification. Proceedings of the 2017 2nd International Conference on Image, Vision and Computing (ICIVC 2017).

